# Cardiovascular Risk of Nonsteroidal Anti-Inflammatory Drugs: An Under-Recognized Public Health Issue

**DOI:** 10.7759/cureus.1144

**Published:** 2017-04-08

**Authors:** Zoltan Varga, Syed rafay ali Sabzwari, Veronika Vargova

**Affiliations:** 1 Internal Medicine Residency, Florida Hospital Orlando; 2 Translational Research Institute

**Keywords:** non-steroidal anti-inflammatory drugs, cardiovascular risk, adverse effect, cardiovascular event, arterial hypertension, heart failure

## Abstract

Nonsteroidal anti-inflammatory drugs (NSAIDs) are drugs with analgesic, anti-inflammatory, and antipyretic activity. Their effect is achieved by the reduction in synthesis of prostanoids. Inhibition of prostanoids is responsible for a substantial risk of adverse effects. The risk of side effects affecting the gastrointestinal tract and kidneys has long been known. The possibilities of blood pressure elevation and the development of congestive heart failure are also widely recognized. Increased incidence of acute myocardial infarction in clinical trials with rofecoxib drew attention to the potential cardiotoxicity of selective cyclooxygenase-2 inhibitors, and similarly, concerns have been raised regarding the cardiovascular safety of non-selective NSAIDs. The safety of NSAIDs with regards to cardiovascular events has been studied in recent years in a large number of retrospective and prospective clinical studies and meta-analyses. The results indicate that cardiotoxicity is a class effect, but the magnitude of the risk is widely variable between individual NSAID drugs. This article aims to summarize the available data on the risk of adverse cardiovascular events with NSAIDs, the clinical impact of these events and possible underlying mechanisms.

## Introduction and background

Nonsteroidal anti-inflammatory drugs (NSAIDs) have numerous serious, potentially life-threatening adverse drug reactions (ADR), yet they belong to the most widely prescribed/used medicines worldwide [1]. Because of a large number of patients exposed to NSAIDs, their side effects represent a serious public health problem.

During therapy with NSAIDs, the patient is at risk of gastrointestinal and renal toxicity, which have long been known [2-3]. Increase in arterial blood pressure (BP) during the administration of NSAIDs and the risk of heart failure exacerbation were also described decades ago [4-5]. The end of the 20th and beginning of the 21st century witnessed multiple large, randomized clinical trials organized to quantify the gastrointestinal risk of the then new group of selective cyclooxygenase-2 (COX-2) inhibitors (coxibs). The Vioxx Gastrointestinal Outcomes Research (VIGOR) study unexpectedly discovered an increased incidence of myocardial infarction (MI) in patients treated with the selective cyclooxygenase-2 inhibitor rofecoxib, compared to naproxen [6]. The results of VIGOR and later the results of multiple other prospective and retrospective studies have prompted a gradual reassessment of the risk-benefit profile of NSAIDs in patients with cardiovascular disease.

The following paper summarizes the available data on the cardiovascular risk of NSAIDs, their potential clinical impact and the possible mechanisms responsible for the increased incidence of cardiovascular events seen with NSAID therapy.

## Review

### The cyclooxygenase enzyme and its physiologic roles

NSAIDs are cyclooxygenase (COX) inhibitors. COX is an enzyme, which produces prostaglandin H2 (PGH-2) from arachidonic acid. PGH-2 is a metabolite converted into prostanoids (prostaglandins, prostacyclins and thromboxanes) by tissue specific enzymes.

Two basic isoforms of cyclooxygenase are known to date: COX-1 and COX-2. Initially, COX-1 was thought to be the constitutive form of the enzyme playing an important role in physiologic functions of the human body. At the same time, COX-2 was considered strictly inducible and thought to be responsible for inflammation and pain under pathologic circumstances. Recently, this theory was proven to be too simplistic. It is now known that COX-2 is permanently present in several tissues of the human body and plays an important role in multiple physiologic processes.

With regards to the most common serious ADRs of NSAIDs, it is important to understand the role of COX-1 in the formation of protective prostaglandin E2 (PGE-2) and prostacyclin (PGI-2). Both of these play a protective role in the gastric mucosa. In thrombocytes, COX-1 forms thromboxane A2 (TXA-2), which is a prostanoid antagonizing the anti-thrombotic and vasodilating effect of PGI-2 formed in the arteries by both COX isoforms. Within the kidney, PGE-2 formed by COX-1 plays a decisive role in the regulation of glomerular filtration, while PGI-2 produced by COX-2 affects renin secretion. Finally, products of both COX isoforms play a role in the kidney regulating excretion of sodium and water.

Based on their selectivity for isoforms of COX, NSAIDs are classified into non-selective cyclooxygenase inhibitors, preferential COX-2 inhibitors and selective inhibitors of COX-2 (coxibs) (Table 1).

**Table 1:** Groups of common NSAIDs according to COX selectivity

Non-selective COX-1 and COX-2 inhibitors:	acetylsalicylic acid, ibuprofen, diclofenac, ketoprofen, indomethacin, naproxen
Preferential COX-2 inhibitors:	meloxicam, nimesulide, etodolac
Selective COX-2 inhibitors:	celecoxib, rofecoxib, etoricoxib, valdecoxib

### Adverse effects involving the digestive and urinary system

Treatment with NSAIDs can result in a wide variety of side effects (Table 2). Soon after the introduction of the first NSAID, acetylsalicylic acid (ASA) to clinical practice it was clear that although effective in treatment of pain and inflammation, ASA had the potential to damage gastric mucosa. Since that time, formation of gastric ulcers has been the most feared complication of NSAID therapy with a generally high perception of risk in the healthcare community.

**Table 2:** Overview of common adverse effects of NSAIDs

Gastrointestinal	erosions and ulcers of gastric mucosa, nausea, vomiting, bloating, diarrhea, constipation
Renal	reduced glomerular filtration rate, Na and water retention, pitting edema, hyperkalemia, kidney failure, interstitial nephritis
Cardiovascular	thrombotic events, increased blood pressure, congestive heart failure, palpitations
Central nervous system	headache, fatigue, insomnia, vertigo, seizures
Other	bleeding, asthma attacks, Reye's syndrome, urticaria, neutropenia

In a retrospective case-control study of 1,457 patients with gastrointestinal (GI) bleeding and 10,000 controls, Garcia-Rodriguez and Jick [7] found a relative risk (RR) of bleeding from an upper gastrointestinal (GI) source to be 4.7 with NSAID therapy. MacDonald, et al. [8] in a prospective, cohort study found the RR of hospitalization for upper GI problems to be 3.94 in a group of 52,293 patients taking NSAIDs. This increased risk persisted throughout the whole duration of treatment and although slowly decreasing with time, remained above baseline even a full year after discontinuation.

COX-1 blockade damages the integrity of the gastric lining. The discovery of COX-2 gave the pharmaceutical industry a theoretical chance to increase the GI safety of analgesic therapy by development of selective COX-2 inhibitors (coxibs). Coxibs were expected to provide analgesic and anti-inflammatory therapy with lower incidence of severe GI side effects. This hypothesis was confirmed in several randomized clinical trials. The VIGOR study compared the risk of GI events in patients on rofecoxib with naproxen. The reason for NSAID treatment in this study was rheumatoid arthritis. The risk of the GI primary outcome was lower in the case of rofecoxib (RR = 0.5, p = 0.001) [6].

Non-selective cyclooxygenase inhibitors can cause a reduction in glomerular filtration rate (GFR), retention of sodium, potassium and water with development of peripheral edema and hypertension (HTN) [9]. Preferential and selective inhibitors of COX-2 have the potential to cause retention of sodium, potassium and water, but the GFR is less impacted by them [10]. Overall, the incidence of renal adverse events among NSAID users is around 18%, however, serious ADRs with clear symptomatology affect one percent of patients [11].

### Thrombotic events

After introduction of the first coxibs to clinical practice, multiple large, randomized clinical trials were organized to assess their gastrointestinal tolerability. Several of them unexpectedly showed an alarming increase in the risk of thrombotic events. The VIGOR study was aimed at evaluating the gastrointestinal safety of rofecoxib and included 8076 volunteers suffering from rheumatoid arthritis. Patients with active, severe cardiovascular disease were not included, and the preventive administration of low-dose ASA was not allowed. The participants were randomized to two arms: one was administered rofecoxib, the other naproxen. The RR of cardiovascular (CV) events with rofecoxib versus naproxen was 2.37 (1.39- 4.06), p = 0.0016 [12].

Similarly, the Adenomatous Polyp Prevention on Vioxx (APPROVe) study, in which rofecoxib was compared to placebo found an increased incidence of thrombotic events, particularly myocardial infarction and stroke in the rofecoxib arm (1.5 vs 0.78 cases per 100 patient years, RR = 1.92, p = 0.008). The relative risk difference became significant 18 months after the start of drug administration [13].

The Celecoxib Long-term Arthritis Safety Study (CLASS) trial compared the safety of celecoxib (400 mg twice/day) with two widely used traditional NSAIDs, ibuprofen and diclofenac. The number of cardiovascular events across all arms was similar with no statistically significant differences. It is important to note that, contrary to VIGOR, 20% of patients were taking a preventative dose of ASA. Rheumatoid arthritis was present in 27% of participants, while the rest required NSAIDs for pain due to osteoarthritis [14].

Several theories were formed in an effort to explain the conflicting results of large clinical trials with rofecoxib and celecoxib. According to the first published theory, the difference in the number of thrombotic events between the arms in VIGOR was not caused by a negative effect of rofecoxib, but by a cardioprotective effect of naproxen. Some studies have indeed shown a moderate protective effect with naproxen, but it appears that the difference between naproxen and rofecoxib in the VIGOR study was too large to be explained only by naproxen-mediated protection [15].

The different results of VIGOR and CLASS might also be explained by a higher baseline cardiovascular risk present in patients in VIGOR (rheumatoid arthritis vs mostly osteoarthritis in CLASS). Baseline CV risk of patients appears to be an important predictor of risk of cardiovascular adverse effects of coxibs [16]. Another possible confounding factor is the use of low dose aspirin by some of the subjects in CLASS. This seems unlikely though, because the difference in risk of CV events between celecoxib and traditional NSAID users was not found to be significant in a post-hoc analysis of subjects without aspirin use [14].

One of the most accepted theories explains the difference by the higher COX-2 selectivity of rofecoxib. In theory, this would cause a more pronounced disbalance between thrombogenic and anti-aggregatory prostanoids. On the other hand, the whole body of evidence gathered from all clinical studies ever conducted with various coxibs and NSAIDs show that increasing selectivity is not always linked to increasing CV risk. Apparently there must be other factors that come into play. Pharmacokinetic and pharmacodynamic characteristics of individual NSAIDs might be more important than initially expected. Celecoxib has a shorter half-life than rofecoxib that theoretically might allow the regeneration of vascular prostacyclin at the end of the dosing interval. Also, the influence of blood pressure cannot be underestimated. The Framingham study showed it to be an important predictor of CV complications. Celecoxib affects blood pressure less than rofecoxib and the exact mechanism leading to this difference is unknown. The reason might lie in the suppressive effect of high dose rofecoxib on degradation of aldosterone. Additionally, celecoxib seems to be a weak inhibitor of carbonic anhydrase [17]. The above described different effects on blood pressure could have far-reaching implications for long-term therapy, though the consequences of short-term treatment, lasting only weeks to a few months are questionable.

After CLASS, several studies comparing the efficacy and safety of celecoxib with placebo or nonselective NSAIDs were organized. Solomon, et al. [16] analyzed six randomized, placebo-controlled trials. The meta-analysis included the trials attention placebo control (APC), prevention of colorectal sporadic adenomatous polyps (PreSAP), alzheimer's disease anti-inflammatory prevention trial (ADAPT), the megestrol acetate (MA-27), celecoxib diabetic macular edema (CDME) and celecoxib/selenium (7950 patients in total with 16,070 patient-years). Based on the results, it appears that the risk of cardiovascular adverse events during treatment with celecoxib depends on two factors: a) baseline cardiovascular risk of patients and b) the dose of the drug used. Particularly important, is the interaction of these factors, which means enhancement of the effect of celecoxib dose in high-risk people. Compared with placebo, the relative risk for all doses combined was found to be 1.6. More interesting is the risk with each individual dosage regimen studied: 400 mg 1x/day- RR = 1.1; 200 mg 2x/day- RR = 1.8; 400 mg 2x/day- RR = 3.1. Unfortunately, the study did not include the regimen 200 mg 1x/day, although currently 80 - 90% of patients on celecoxib are receiving this regimen. Recently, the results of the long awaited Prospective Randomized Evaluation of Celecoxib Integrated Safety versus Ibuprofen or Naproxen trial (PRECISION) was published [18]. The study compared 100-200 mg celecoxib two times a day with 600-800 mg ibuprofen three times a day and 375-500 mg naproxen two times a day. The subjects suffered from osteoarthritis or rheumatoid arthritis and at the same time were at increased risk for CV events. Celecoxib was found to be non-inferior to ibuprofen and naproxen for the primary outcome of a composite of CV death, non-fatal myocardial infarction (MI)cor non-fatal stroke.

Clinical studies of newer coxibs also brought distressing results. Therapeutic Arthritis Research and Gastrointestinal Event Trial (TARGET) compared lumiracoxib with naproxen and ibuprofen. In patients not on low dose ASA, cardiovascular events occurred more frequently in the selective COX-2 group, but the difference was not statistically significant (0.26 vs. 0.18 per 100 patient-years, RR = 1.22, p = 0.43). The study group consisted of patients with osteoarthritis [19]. In another study enrolling patients after coronary artery bypass grafting (CABG), a series of events affecting the cardiovascular system emerged, ultimately leading to rejection of parecoxib (prodrug of valdecoxib) by the food and drug administration (FDA) [20]. A trial with valdecoxib itself, after CABG showed a similarly unfavorable CV safety profile: RR = 3.08 [21]. An increased number of cardiovascular events compared with placebo and with traditional NSAIDs was also reported in the case of etoricoxib [22]. On the other hand, in the recently published retrospective analysis by de Souza Brito, et al. [23], no elevation of CV event risk was present in post-CABG patients receiving NSAIDs. The study included a robust number of patients. On the other hand, all NSAIDs were grouped together without specifying the actual medications used, which poses a significant limitation.

After the discovery of increased risk of thrombotic events with COX-2 inhibitors, scientists focused their efforts on ruling out or confirming similar ADRs of non-selective NSAIDs. Therefore in recent years, the cardiovascular safety of NSAIDs has been addressed in many clinical trials and meta-analyses.

Trelle, et al. [24] included in their meta-analysis each randomized and controlled study comparing NSAIDs with placebo or with other NSAIDs and with monitoring lasting for at least 100 patient-years. These conditions were met by 31 studies, involving a total of 116,429 patients with over 115,000 patient-years of follow-up. The primary outcome was acute myocardial infarction (AMI). As secondary outcomes, stroke, CV death and death from any cause were studied. Results were statistically evaluated for three non-selective NSAIDs (naproxen, ibuprofen, diclofenac) and four coxibs (rofecoxib, celecoxib, lumiracoxib, etoricoxib). The highest risk of AMI compared to placebo was found in rofecoxib (RR = 2.12) and lumiracoxib (RR = 2.00), from non-selective drugs for ibuprofen (RR = 1.61). Ibuprofen and diclofenac were associated with the highest risk of stroke (RR = 3.36 and 2.86 respectively) (Table 3). Another meta-analysis of randomized trials found RR of major coronary events to be 1.76 (1.31-2.37; p=0.0001) for coxibs combined, 1.7 (1.19-2.41; p=0.0032) for diclofenac and 2.22 (1.1-4.48; p=0.0253) for ibuprofen [25].

**Table 3:** Overview of the results of Trelle, et al. comparing the risk of cardiovascular events with NSAIDs to placebo or other NSAIDs

	Myocardial infarction (RR)	Stroke (RR)	Cardiovascular death (RR)	Death (RR)
Naproxen	0.82 (0.37-1.67)	1.76 (0.91-3.33)	0.98 (0.41-2.37)	1.23 (0.71-2.12)
Ibuprofen	1.61 (0.50-5.77)	3.36 (1.00-11.60)	2.39 (0.69-8.64)	1.77 (0.73-4.30)
Diclofenac	0.82 (0.29-2.20)	2.86 (1.09-8.36)	3.98 (1.48-12.70)	2.31 (1.00-4.95)
Celecoxib	1.35 (0.71-2.72)	1.12 (0.60-2.06)	2.07 (0.98-4.55)	1.50 (0.96-2.54)
Etoricoxib	0.75 (0.23-2.39)	2.67 (0.82-8.72)	4.07 (1.23-15.70)	2.29 (0.94-5.71)
Rofecoxib	2.12 (1.26-3.56)	1.07 (0.60-1.82)	1.58 (0.88-2.84)	1.56 (1.04-2.23)
Lumiracoxib	2.00 (0.71-6.21)	2.81 (1.05-7.48)	1.89 (0.64-7.09)	1.75 (0.78-4.17)

The increased risk of thrombotic events mediated by non-selective NSAIDs and selective COX-2 inhibitors has been also shown by large-scale epidemiological studies. The results of 23 controlled observational studies (six cohort and 17 case-control) were summarized by McGettigan and Henry in their meta-analysis [26]. The case-control studies included 86,193 patients with cardiovascular events and 528,000 controls. In the cohort studies, 75,520 coxib users and 375,619 users of nonselective NSAIDs were compared to 594,720 patients without exposure to NSAIDs. The resulting RR values of cardiovascular events are shown in Tables 4-5. From non-selective NSAIDs, the highest risk was present in the case of diclofenac (RR = 1.40) and the safest was naproxen (RR = 0.97). For rofecoxib (RR = 1.35), the risk was significantly influenced by the dose.

**Table 4:** Relative risk of cardiovascular events with non-selective NSAIDs compared to controls in a meta-analysis (McGettigan and Henry)

	Non-selective NSAIDs					
	Naproxen	Diclofenac	Ibuprofen	Indomethacine	Piroxicam	Non-selective NSAIDs combined
Case-control studies	0.96(0.84-1.10)	1.36(1.21-1.54)	1.06(0.95-1.18)	1.30(1.07-1.60)	1.06(0.70-1.59)	1.10(0.98-1.24)
Cohort studies	0.94(0.85-1.04)	1.36(0.51-3.65)	1.12(0.90-1.38)	--	--	1.10(0.95-1.29)
Case-control and cohort studies combined	0.97(0.87-1.07)	1.40(1.16-1.70)	1.07(0.97-1.18)	1.30(1.07-1.60)	1.06(0.70-1.59)	1.10(1.00-1.21)

**Table 5:** Relative risk of cardiovascular events with preferential and selective COX-2 inhibitors compared to controls in a meta-analysis (McGettigan and Henry)

	Preferential and selective inhibitors of COX-2				
	Meloxicam	Celecoxib	Rofecoxib ≤ 25 mg/day	Rofecoxib ˃ 25 mg/day	Rofecoxib combined
Case-control studies	1.25(1.00-1.55)	1.01(0.90-1.13)	1.21(1.08-1.36)	1.89(1.43-2.51)	1.31(1.18-1.46)
Cohort studies	--	1.22(0.69-2.16)	1.51(0.73-3.13)	2.46(1.29-4.71)	1.53(0.68-3.44)
Case-control and cohort studies combined	1.25(1.00-1.55)	1.06(0.91-1.23)	1.33(1.00-1.79)	2.19(1.64-2.91)	1.35(1.15-1.59)

A cohort study focused on the risk of death from coronary causes and AMI in NSAID users after hospitalization for acute coronary syndrome. The cohort included 48,566 patients, of which 19,253 were taking NSAIDs. Patients were followed for more than 111,000 patient-years. The results showed significant risk, especially in the first 90 days of NSAID use. The RR of AMI and death from coronary causes during analgesic therapy lasting less than 90 days: naproxen- 0.88, ibuprofen- 1.67, diclofenac- 1.86, celecoxib- 1.37, rofecoxib- 1.46 [27].

### Possible mechanisms for the increased risk of thrombotic events

The first theory trying to explain the risk of thrombotic events of selective COX-2 inhibitors was based on the assumption that COX-2 in the vessel wall was an important source of prostacyclin, whereas COX-1 was responsible for the production of thromboxane in platelets. Selective COX-2 inhibition would in this situation decrease the production of PGI-2, which is a vasodilating and antithrombotic substance, without inhibiting the vasoconstricting and thrombogenic TXA-2. The resulting imbalance between pro-thrombotic and antithrombotic factors could lead to thrombosis [28].

On the other hand, the greater risk of thrombotic events with highly selective COX-2 inhibitors when compared with non-selective NSAIDs apparently cannot be explained only by the concomitant inhibition of platelet function with the non-selective agents. For a clinically significant suppression of platelet aggregation, greater than 95% inhibition of COX-1 is needed. This happens only with aspirin, and in certain situations with high dose naproxen [29].

The overall strength of COX-2 inhibition appears to be the most important determinant of cardiovascular risk [29]. Results of numerous experiments show the important role of COX-2 in production of prostacyclin in the vascular bed [30]. The inhibition of PGI-2 production causes an increase in vascular tone, blood pressure elevation, thrombogenic state and likely atherosclerosis. Cyclooxygenase-2 and PGI-2 also seem to play a key role in the resistance of the myocardium to ischemic-reperfusion injury observed after ischemic preconditioning or after administration of certain medications [31-32]. The potential importance of cardioprotective effects of COX-2 was also underscored in the position paper written by the working group for cardiovascular pharmacotherapy of the European Society of Cardiology [33].

Another contributing factor in the CV toxicity of NSAIDs might be the inhibition of cardioprotective effect of statins. Administration of statins before ischemia leads to reduction in the size of myocardial necrosis in animal models of ischemia-reperfusion injury. Selective COX-2 inhibition attenuates this statin-induced cardioprotection [32-34]. In their study, Birnbaum, et al. [35] showed that high dose aspirin leads to sufficient COX-2 inhibition to reduce the cardioprotective effect of statins. In a study on healthy volunteers, it was demonstrated that administration of rosuvastatin before forearm ischemia results in a reduction of endothelial dysfunction induced by ischemia and reperfusion and that the beneficial effect of the statin disappears if celecoxib is co-administered [36].

Guidelines for the management of various forms of coronary artery disease recommend administration of statins to normocholesterolemic patients at high risk of coronary events [37]. The clinical benefit of statin therapy in this situation might at least partially be mediated by its pleiotropic effects. Patients with coronary artery disease are mostly elderly and often suffering from musculoskeletal conditions with the need to use NSAIDs. Further studies are therefore needed to clarify the potential interactions between statins and NSAIDs in this scenario.

Despite of the above described theories, the exact mechanisms responsible for the high variability of the thrombotic risk with various NSAIDs remain unknown. To further elucidate the reason behind the differences in the observed degree of CV risk between various NSAIDs, more focus should be placed on studies scrutinizing their exact pharmacokinetic and pharmacodynamic properties [38].

### Increase in arterial blood pressure

NSAIDs can cause sodium and water retention, as well as reduce formation of the vasodilator prostacyclin in the vessel wall. The risk of increased blood pressure during treatment with NSAIDs has long been known. This side effect seems to be present in all NSAIDs except low dose ASA, although some NSAIDs appear to be safer than others [39]. According to a meta-analysis by Johson, et al. [40], analgesics from the group of NSAIDs lead to an elevation in mean blood pressure by 5 mmHg, but in this study, the increase in blood pressure reached the level of statistical significance only in the subgroup with medically controlled hypertension.

The blood pressure increasing effect is present also in the case of selective COX-2 inhibitors. According to a meta-analysis by Chan, et al. [41], the risk of blood pressure increase is higher with coxibs than with non-selective NSAIDs (RR of a BP increase: coxibs vs. placebo: 1.49, p=0.04; coxibs vs. non-selective NSAIDs: 1.12, p=0.23). The same study highlighted the differences between various coxibs. Significant increases in blood pressure were found with rofecoxib and etoricoxib, while the effect of celecoxib on blood pressure appeared to be minimal.

The risk of NSAID-mediated BP increase in patients with pharmacologically controlled hypertension also depends on the antihypertensive drug used by the patient. The effect of beta-blockers and various renin-angiotensin-aldosterone system (RAAS) inhibitors seems to be influenced the most [40,42]. The most likely reason is that NSAIDs under normal circumstances inhibit the production of renin as a compensatory mechanism for the retention of water and sodium caused by them and also by direct COX-2 inhibition. Thus, under normal circumstances, NSAIDs cause a decrease in secretion of renin, which counteracts the hypertensive potential of NSAID analgesics. If RAAS is inhibited chronically by the antihypertensive regimen used by the patient, the previously described compensatory mechanism can't help to avoid an increase in the blood pressure [43].

Most patients with severe rheumatoid arthritis and osteoarthritis are elderly and often have multiple co-morbidities, including hypertension treated with antihypertensive drugs. Under these circumstances the safe and effective treatment of their symptoms is problematic [44]. In a pilot study by Varga, et al. enrolling 428 patients taking NSAIDs during hospitalization at the department of internal medicine of a district hospital in Slovakia, 58.2% of the NSAID using patients received concomitant antihypertensive medications, most commonly drugs that depressed the activity of RAAS [45].

### Heart failure

Inhibition of prostanoid production in the kidney may reduce glomerular filtration and excretion of sodium and water. NSAIDs are therefore associated with risk of hypervolemia and worsening heart failure. The risk is increased in patients with impaired renal or cardiac function and it’s the highest if pre-existing congestive heart failure (CHF) is present, especially if controlled by diuretics [46-47].

The study by Heerdink, et al. [46] showed a RR of hospitalization for heart failure to be 1.8 (95% CI= 1.4-2.4), when NSAIDs were administered to patients treated with diuretics. The authors did not find a significant difference between individual NSAIDs, which suggests a class effect. The highest risk of heart failure decompensation was present within the first days of treatment initiation and gradually decreased to the level of placebo after a month.

The risk of hospitalization for heart failure while on NSAIDs was studied also by Page and Henry [47]. NSAID users were found to have a relative risk of 2.1 when compared to non-users. In patients with established CV disease, the RR was noticeably higher (10.5). According to estimates by the authors, NSAID use might play a role in up to 19% of cases of newly diagnosed congestive heart failure.

Mamdani, et al. [48] compared in their cohort study the risk of hospitalization for congestive heart failure in patients treated with coxibs, non-selective NSAIDs and controls. The most significant risk was found in patients on rofecoxib (RR = 1.8, 95% CI= 1.5-2.2). The relative risk in users of non-selective agents was 1.4 (95% CI= 1.0-1.9). Celecoxib was not associated with increased risk.

The perception of risk of CHF exacerbation caused by NSAIDs seems to be higher in the medical community than the perception of risk of other CV adverse effects, since according to new data, patients with CHF are less likely to get an NSAID prescribed then patients with other forms of CV disease [49].

## Conclusions

The results of both interventional and observational studies point towards the increased CV risk being a class effect of NSAIDs. The level of risk displays a large variability between individual drugs in the group and seems to be affected by the baseline cardiovascular risk of patients, although CV ADRs can develop even in individuals without a pre-existing CV condition. It appears that the absolute degree of COX-2 inhibition and not COX-2 selectivity is responsible for the increased risk. Differences in pharmacokinetics might also play a role.

NSAIDs can lead to an elevation in blood pressure, especially in patients treated with drugs inhibiting RAAS. The risk of blood pressure elevation shows a large variability between individual NSAIDs. Patients with congestive heart failure are at risk of the disease decompensation while taking NSAIDs. The risk is highest in patients taking diuretics, especially during the first few weeks of NSAID treatment.

In conclusion, it should be stressed that candidates for long-term administration of NSAIDs are commonly elderly patients, who often suffer from arterial hypertension, CHF and coronary artery disease. Chronic NSAID users are therefore very commonly at elevated risk of cardiovascular adverse effects. Adequate monitoring for signs and symptoms of adverse effects with proper patient education are required to increase patient safety during NSAID therapy. The duration of NSAID treatment should be limited as much as the clinical situation allows and only the minimal effective dose should be used.
